# A systematic review exploring the content and outcomes of interventions to improve psychological safety, speaking up and voice behaviour

**DOI:** 10.1186/s12913-020-4931-2

**Published:** 2020-02-10

**Authors:** Róisín O’Donovan, Eilish McAuliffe

**Affiliations:** UCD School of Nursing, Midwifery & Health Systems, UCD Health Sciences Centre, University College Dublin, Dublin 4, Ireland

**Keywords:** Psychological safety, Speaking up, Voice behaviour, Teams, Interventions

## Abstract

**Background:**

Having psychologically safe teams can improve learning, creativity and performance within organisations. Within a healthcare context, psychological safety supports patient safety by enabling engagement in quality improvement and encouraging staff to speak up about errors. Despite the low levels of psychological safety in healthcare teams and the important role it plays in supporting patient safety, there is a dearth of research on interventions that can be used to improve psychological safety or its related constructs. This review synthesises the content, theoretical underpinnings and outcomes of interventions which have targeted psychological safety, speaking up, and voice behaviour within a healthcare setting. It aims to identify successful interventions and inform the development of more effective interventions.

**Methods:**

A key word search strategy was developed and used to search electronic databases (PsycINFO, ABI/Inform, Academic search complete and PubMed) and grey literature databases (OpenGrey, OCLC WorldCat, Espace). Covidence, an online specialised systematic review website, was used to screen records. Data extraction, quality appraisal and narrative synthesis were conducted on identified papers.

**Results:**

Fourteen interventions were reviewed. These interventions fell into five categories. Educational interventions used simulation, video presentations, case studies and workshops while interventions which did not include an educational component used holistic facilitation, forum play and action research meetings. Mixed results were found for the efficacy or effectiveness of these interventions. While some interventions showed improvement in outcomes related to psychological safety, speaking up and voice, this was not consistently demonstrated across interventions. Included interventions’ ability to demonstrate improvements in these outcomes were limited by a lack of objective outcome measures and the ability of educational interventions alone to change deeply rooted speaking up behaviours.

**Conclusion:**

To improve our understanding of the efficacy or effectiveness of interventions targeting psychological safety, speaking up and voice behaviour, longitudinal and multifaceted interventions are needed. In order to understand whether these interventions are successful, more objective measures should be developed. It is recommended that future research involves end users in the design phase of interventions, target both group and organisational levels, ensure visible leader support and work across and within interdisciplinary teams.

**Prospero registration number:**

CRD42018100659.

## Background

When teams are psychologically safe, there is a shared belief that members are safe to take interpersonal risks, such as speaking up and engaging in voice behaviour. This definition of psychological safety was proposed by Amy Edmondson [1] in 1999 and began research on psychological safety as a phenomenon that exists at a group level and is built through workplace interactions. Psychological safety is a key determinant of high-quality communication, trust and decision making which improves team performance and, therefore, plays an important role within workplace teams [2–4]. Psychological safety plays a particularly vital role in high-risk work contexts, such as healthcare [3]. When healthcare teams are psychologically safe they are more likely to engage in quality improvement and team learning initiatives [5, 6]. This engagement allows healthcare teams to deal with the increased knowledge they need to absorb, the specialisation of healthcare professionals and the resulting interdependence between these professionals [5]. Therefore, having psychologically safe teams is critical to the delivery of safe and effective care within a complex, dynamic and high stakes work environment. However, a culture of fear and low psychological safety still exists within healthcare organisations [7–9]. Given the important outcomes associated with psychological safety, there is a need to develop and implement interventions to improve psychological safety within healthcare teams. This article will build on previous reviews of psychological safety literature [2, 3] by examining interventions which specifically aimed to improve psychological safety, or its related constructs, speaking up and voice behaviour. It is hoped that the findings of this synthesis will inform the development of future interventions.

Although research to date has illustrated the beneficial outcomes of psychological safety, there is little guidance on how teams can introduce, improve and maintain psychological safety. In their cross-industry comparison study examining psychological safety in both healthcare and educational contexts, Edmondson and colleagues [10] acknowledge that there is limited research on interventions to promote psychological safety. They argue that psychological safety would be a useful focus for interventions and provide suggestive avenues for research into such interventions. However, given the dearth of interventions targeting psychological safety, this review will take a broader view by including interventions targeting speaking up and voice behaviour, which are closely related to psychological safety.

Speaking up and voice are interpersonally risky behaviours which are facilitated by psychological safety [5, 11–13]. Lack of psychological safety has been associated with silence [14]. Even when employees believe they have something useful to say, lack of psychological safety often leads them to choose silence over voice [9, 15–17]. It is necessary to encourage an organisational climate where it is safe to speak up and voice ideas or concerns, as this enables organisational learning and organisational safety [9, 17, 18].

This review aims to identify team building interventions which have focused on psychological safety and its related components, speaking up, voice and silence behaviours. Team development interventions have been broadly defined as intentional actions which attempt to improve or support teams that may be struggling or adequately performing or maximise the capacities of teams ready to advance to a higher level of performance [19]. These interventions are relevant to this review, because they are suited to targeting psychological safety, speaking up and voice behaviours. They focus on interpersonal relations in order to increase teamwork process and emergent states such as mutual support and communication [20]. Team development interventions can also focus on problem solving which promotes synergy through encouraging team members to practice setting goals, developing interpersonal relations, clarifying team roles and working to improve organisational characteristics through participating in problem solving tasks. These types of team development interventions have the strongest and most consistent effects on affectively driven states that are critical to teams, such as psychological safety [20].

This systematic review of the literature will synthesise the content, theoretical underpinnings and outcomes of interventions which have been conducted to date to improve psychological safety and its related components, speaking up and voice behaviour, within a healthcare setting. Both efficacy and effectiveness outcomes will be considered. This review aims to answer the research questions: *What interventions have been conducted to improve psychological safety, speaking up and voice behaviour within a healthcare setting? What are the underlying theoretical approaches in these interventions? How have these interventions been evaluated? Which interventions have been most effective for encouraging a climate of psychological safety?* This will enable future research to build on what has been done before to create a reliable intervention for improving psychological safety in workplace teams.

## Methods

A systematic review was used to explore the above research questions. Systematic reviews are an essential tool for synthesising the evidence from available studies to answer a specific research questions [21–24]. The Cochrane and Preferred Reporting Items for Systematic Reviews and Meta-Analyses (PRISMA) guidelines [23–25] have been followed in this review.

The protocol for this review has been published on Prospero (registration number: CRD42018100659). Since publishing this protocol, the following changes were made to the reivew:

- The terms Speaking Up and Voice Behaviour were added to the title of the review in order to accurately capture the interventions reviewed. Originally, this review intended to examine interventions targeting psychological safety alone. However, given the limited number of interventions targeting psychological safety, the inclusion criteria were widened to include interventions targeting speaking up and voice behaviour.
- After conducting the search, a large number of interventions conducted within a healthcare setting were identified. In addition, the literature highlighted the import role played by psychological safety, speaking up and voice in a healthcare setting. Therefore, the inclusion criteria for the setting of the interventions was narrowed from “no defined setting” to “within a healthcare setting”. As well as reflecting the body of literature found by this review, narrowing the setting allowed the review to explore the identified interventions in more detail and to consider their impact within a specific work setting.
- The research question “how have these interventions been evaluated?” was added to the final version of this review in order to explore the ways in which each study assessed the impact of the intervention on psychological safety, speaking up and voice.
- The inclusion criteria of the final review were updated to reflect the above changes.

### Inclusion and exclusion criteria

Studies eligible for inclusion were peer reviewed, from any country, published between 1999 and 2018 and explored the development, implementation and/or evaluation of interventions relevant to psychological safety in healthcare settings. Given the limited number of interventions targeting psychological safety, the inclusion criteria were widened to include interventions targeting speaking up and voice behaviour.

Studies were excluded if they were not available in English or if they reported on interventions conducted outside healthcare settings.

### Search strategy

The search strategy used key words identified through a scoping review of the literature. They were grouped together using the OR Boolean term. The resulting search strategy was reviewed by a researcher with extensive systematic review experience. The final search strategy was: “Psychological* safe*” OR “Speak* up” OR voic* OR silen*. The term “intervention” had been included in an earlier iteration but was excluded because it narrowed the search too much. A full search strategy can be found in Additional file 1.

### Information sources

Electronic databases were searched between the 19th of March 2018 and the 8th of June 2018 to find relevant studies (See search strings in online supplementary material). Electronic databases searched were: PsycINFO, ABI/Inform, Academic search complete and PubMed.

A grey literature search was conducted to supplement the above searches. Grey literature was identified by searching electronic databases which had a broad scope and the ability to conduct specific searches [26, 27]. The databases searched were; OpenGrey, OCLC WorldCAT, Espace (Curtin’s institutional repository). In addition, the authors hand-searched the reference lists of included studies and contacted experts in the field to identify any eligible studies.

### Study screening

Covidence, an online specialised systematic review website, was used to screen records. One reviewer screened titles and abstracts based on the eligibility criteria. When the eligible papers were identified, two reviewers independently reviewed each text. The reviewers met to discuss and resolve any conflicts or disagreements. An option to involve a third reviewer if agreement could not be reached was put in place but proved unnecessary, as following discussion, the original two reviewers reached agreement on all papers for inclusion.

### Data extraction process

A data extraction template was developed to capture the relevant information from included studies. This template was based on the third version of guidelines produced by Cochrane in 2014 for data collection for intervention reviews of randomised control trials and non-randomised control trials and recommendations from Hoffmann and colleagues [28]. Information was collected for: aims, design, theoretical underpinnings, details of the intervention, participant information and outcomes. The final template can be seen in Additional file 2.

### Quality assessment

Depending on the study design, the Critical Appraisal Skills Programme [29] Qualitative Checklist, Cohort Study Checklist, or the Mixed Methods Appraisal Tool [30] were used to assess the quality of included studies.

### Study synthesis

Given the heterogeneity in interventions and measures used in this review, a narrative approach to synthesis was deemed most appropriate [31]. Narrative synthesis relies on words and text to ‘tell the story’ of the included studies [31]. Based on guideline from Popay and colleagues [31], the narrative synthesis followed three iterative steps: organising studies into logical categories by becoming familiar with them, comparing them to one another and synthesising their findings; analysing the findings within each category by exploring relationships within and between the studies and synthesising data under the relevant themes.

## Results

### Search result

The database search yielded 8947 studies and 11 grey literature studies were identified. After 5614 duplicates were removed, 3344 were screened. Three thousand one hundred forty-eight studies were excluded based on title and abstract screening, leaving 196 studies for full text screening. One hundred eighty-two full text articles were excluded, leaving 14 studies included in the review.

**Fig. 1:**
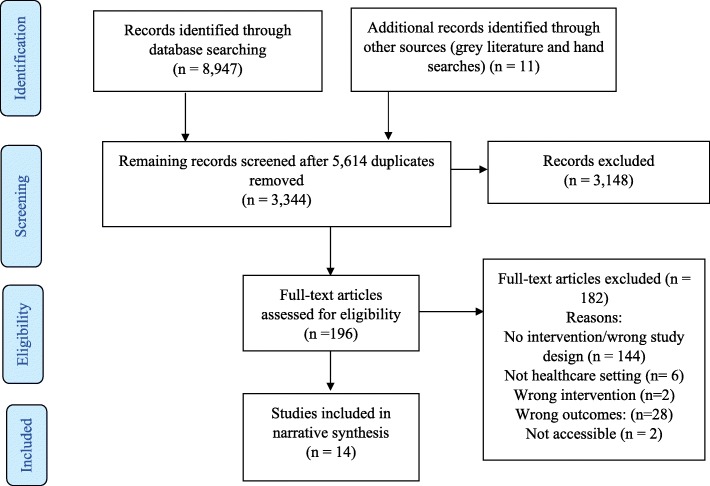
PRISMA flow diagram. This PRISMA flow diagram illustrates the inclusion and exclusion of identified studies

Figure 1 shows the PRISMA flow chart which summarises the screening stage of this review.

### Quality assessment

No study was excluded from the review based on quality assessment (see Additional file 3).

### Included studies

A summary of included studies can be found in Additional file 4: Tables S1, S2, S3, S4 and S5. They were divided into interventions using education and simulation (*n* = 5), interventions using education and leadership videos (*n* = 2), interventions using education and case studies (*n* = 3), interventions using educational workshops alone (*n* = 1) and non-educational interventions (*n* = 3). All interventions are synthesised below.

### Narrative synthesis

Interventions conducted to improve psychological safety or its related components.

#### Educational interventions and simulation exercises

Simulation exercises offer opportunities for developing skills without endangering the health of patients through placing them in situations that replicate real clinical practice [32–34]. The simulation exercises included in five educational interventions presented participants with opportunities to practice speaking up behaviour and were followed by group reflection and debriefing.

Both Pian-Smith and colleagues [35] and Raemer and colleagues [36] used simulation-based scenarios to present anaesthesiologists with opportunities to speak up to healthcare professionals (nurses, surgeons and anaesthesiologists). Both conducted educational workshops which introduced participants to tools for speaking-up (see Additional file 4: Table S1). Pian-Smith and colleagues [35] found improvements in anaesthesiologists speaking up behaviours, however, Raemer and colleagues [36] found no statistically significant changes. Given that Pian-Smith and colleagues [35] explicitly told participants that there would be opportunities to practice speaking up, they had expected their scores to be much higher. Participants in both studies were less likely to speak up to a circulating nurse, suggesting a lack of appreciation of the role of circulating nurses. However, low levels of speaking up were also identified within the anaesthesiologist discipline, with only 25% of participants speaking up to their anaesthesiologist colleague [36].

Dufresne [37] simulated a critical incident for anaesthesia teams. They found that the debriefing leaders’ behaviour had a significant impact on the development of psychological safety. Specifically, when leaders balanced advocacy and inquiry language in the first 10 minutes of the debriefing, the team had lower psychological safety. There were also lower levels of psychological safety when the leader used negative evaluative statements. This suggests that, to cultivate psychological safety, leaders should avoid making early evaluative statements about team or individual performance. Further analysis also suggested when leaders showed they were willing to share their own insights, the team felt more psychologically safe. However, this finding did not reach significance.

Ginsburg and Bain [38] used simulation as part of their multifaceted intervention to promote speaking up behaviour and teamwork in an emergency department (ED). This intervention took place within the context of the hospital’s new Accountability Framework, which holds staff accountable to speak up in the face of unsafe or unprofessional behaviour. Participants were given the opportunity to practice speaking up techniques during role playing simulations and to take part in debriefing sessions, staff huddles and one to one meetings. While there was no significant difference between the ED and Intensive Care Unit (control group) at baseline, the teamwork climate score in the ED was significantly higher post intervention. This score included measures of “speaking up”, but these results were not reported separately.

Thomas and colleagues [39] used simulation to assess changes in team behaviours following an educational intervention. While the intervention focused on a variety of team behaviours, speaking up and voice inquiry, information sharing, and assertion were most relevant to this review. Participants completed a simulated resuscitation where they could use the behaviours they had been taught. Compared to the control group, the intervention group showed more incidents of inquiry, information sharing and assertion.

#### Leaders video presentations

Two educational interventions used video presentations to communicate leaders support for speaking up. O’Connor et al. [40] presented videos of attending physicians discussing situations they faced as interns where their communication and assertiveness skills were challenged. While their intervention had no significant effect on interns’ attitudes towards speaking up about stress or to seniors, the post-training group had significantly more positive attitudes towards speaking up to seniors than the pre-training group. Participants’ speaking behaviour was measured using standardised patient exercises, which showed no significant improvement.

Sayre and colleagues [41] used videos of senior staff expressing their expectation and support for nurses to speak up to remove any implicit sanctions against nurses speaking up. After the videos, participants discussed barriers to speaking up and developed action plans. The intervention group showed a significant improvement in speaking up survey scores and individual lists of nurse behaviours (see Additional file 4: Table S2). There was no difference found in the control group.

#### Video presentations and case studies

Johnson & Kimsey [42] used video presentations of scenarios where there was a risk or an error to spark discussion. Like Pian-Smith [35] and Raemer [36] they introduced tools for speaking up. After the course, the majority (78%) of participants reported believing that they were better able to question decisions or actions of those in authority and were no longer afraid to ask questions (75%). As an objective measure, they found a marked decrease in the number of near misses or sentinel events requiring root cause analysis post training.

Coyle et al. [43] used video dramatization of a medical event and case studies of medical events that occurred in the study clinic to improve attitudes and behaviour related to medical event reporting. They also conducted educational conferences (see Additional file 4: Table S3). According to questionnaires completed post intervention, there was no significant change in participants’ attitude and behaviour towards medical event reporting. However, those who participated in more conferences showed a more positive change in medical event reporting attitudes and behaviour.

Shapiro et al. [44] used video vignettes to encourage clinicians to discuss professional behaviour and the responsibility of bystanders to speak up. Participants were taught specific strategies for managing conflict and speaking up to colleagues who have behaved unprofessionally (see Additional file 4: Table S3). Following this intervention, the number of reported concerns regarding professional behaviour increased across 3 years. Participants also reported that they were aware of their personal role in ensuring a culture of professionalism.

#### Educational workshops

Cave et al. [45] was the only intervention to educational workshops alone. They introduced the CENTRE tool to teams by providing education on the use of the guidelines. CENTRE is a tool which establishes guidelines to promote psychological safety by focusing on confidentiality, equal airtime and non-judgemental listening (see Additional file 4: Table S4). While 17 health care group leaders have said that they found using CENTRE helpful, no formal assessment of this tool has been published to date. Further research is needed to test the effectiveness of this tool in promoting psychological safety.

#### Interventions without educational component

The studies which did not include an educational component all used different interventions and are discussed separately below.

Swahnberg and Wijma [46] used an intervention based on “forum play” (see Additional file 4: Table S5) to understand staffs perceptions of Abuse in Health Care (AHC). Findings were particularly relevant to psychological safety as the intervention created an open climate where all staff felt comfortable discussing AHC. Staff shifted from being detached to having an emotional engagement with AHC. They saw acting against or speaking up about AHC as their responsibility and emphasised the critical role played by bystanders.

Brown and McCormack [47] used holistic facilitation to create psychologically safe spaces where nurses could explore their oppressed behaviours, helping them to discuss differences in opinions more openly within a multidisciplinary team. The facilitation sessions made ward leaders more aware of the role they played in creating a culture of psychological safety in their unit. This enabled the leaders to build trusting partnerships that permitted information and knowledge sharing which could help solve problems.

O’Leary [48] conducted action research meetings with two newly-formed interprofessional project teams. The supportive leadership style used by the author encouraged psychological safety within team meetings. However, psychological safety developed differently in each team. In the first team, a psychologically safe space developed, allowing them to share power and to co-generate knowledge. In the other team, psychological safety did not fully develop. The differences between the teams in this study were explained by the impact of organisational norms and stability in team membership, with organisational norms of shared decision making and a stable core group of team members supporting the development of psychological safety.

#### Interventions most effective at improving psychological safety and its related components

**Fig. 2:**
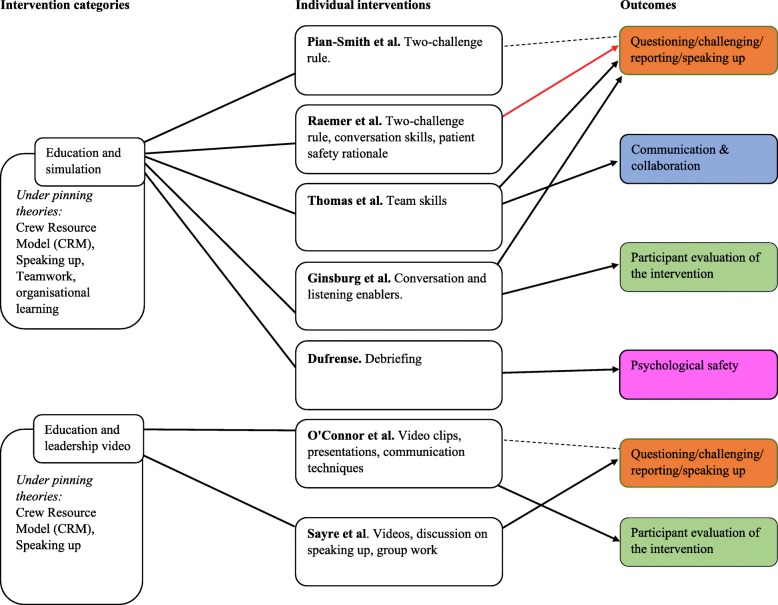

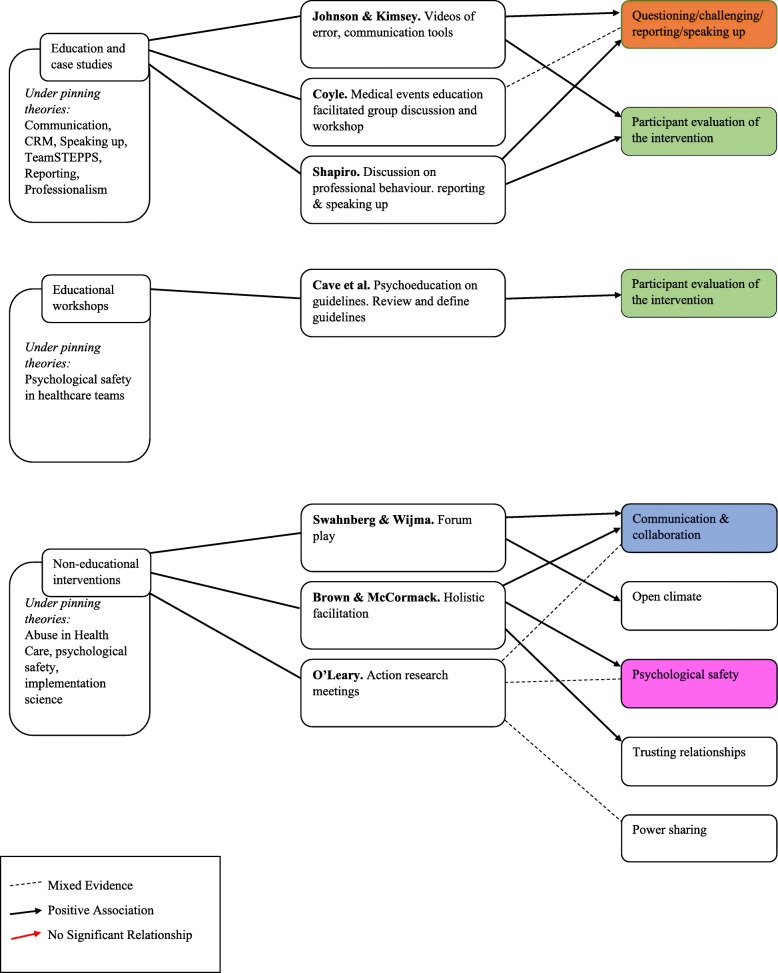
Map of interventions categories, individual interventions review and relevant outcomes. This diagram maps the intervention categories, the studies grouped within them and the relationship between each intervention and key outcomes

The diagram presented in Fig. 2 maps the relationships between the five categories of interventions and outcomes which are relevant to psychological safety.

As can be seen in Fig. 2, mixed results were found for the impact of the interventions on outcomes related to psychological safety, speaking up and voice. Five studies [38, 39, 41, 43, 44] reported improved and three studies [35, 40, 43] found mixed results for “questioning, challenging, reporting or speaking up”. One further study [36] found no significant relationship. Three studies [39, 46, 47] reported improved and one study [48] found mixed results for “communication and collaboration” following interventions. Five studies [38, 40, 42, 44, 45] reported positive evaluations by participants post-interventions. Three studies had “Psychological safety” as an outcome, with one of these reporting mixed results for the impact of the intervention on psychological safety [48] and the other two reporting improvement [37, 47]. Mixed results were found for the impact of one intervention on “power sharing” [48]. Lastly, one study [46] showed a positive impact of the intervention on creating an “open climate” and another showed improvement in “trusting relationships” [47].

## Discussion

This review examined educational and non-educational interventions which targeted psychological safety, speaking up and voice behaviour. The outcomes from these studies were mixed. These mixed results limit our ability to accurately answer the research question: *“Which interventions have been most effective for encouraging a climate of psychological safety?”.* However, the results of this review highlight areas where further research is needed to improve our understanding of the efficacy or effectiveness of interventions targeting psychological safety, speaking up and voice.

### Emerging themes and issues for future research

#### Limitations of educational interventions

Educational interventions identified challenges related to changing deeply rooted speaking up behaviours and questioned whether education alone is sufficient [35, 36]. Implementation science literature suggests that education alone is insufficient for changing behaviour and that it is necessary to have a context which is receptive to change and appropriate facilitation [47, 49]. The limitations associated with educational interventions may explain the mixed outcomes from studies in this review. Some educational studies suggested that there would have been more improvement if interventions had been conducted over a longer period of time [38, 41] and O’Leary [48] highlights how having core team members who are meeting regularly supported the development of psychological safety. These findings suggest that educational interventions may benefit from more regular, longitudinal and multifaceted interventions for improving psychological safety, speaking up and voice.

#### Measuring outcomes

This review identified issues with outcome measurement. Although the simulated scenarios used were as close as possible to reality, they are limited by participants’ awareness that they are not in a real clinical environment. This perception of scenarios realism may affect participants’ behaviour particularly within a healthcare context, when issues of patient safety arise. However, the results from the simulated scenarios made an important contribution to the evidence in this review by providing behavioural evidence of changes relevant to psychological safety, speaking up and voice. This evidence was missing from studies which used questionnaire and survey measures, which were limited by the potential for self-report bias.

According to Shuffler et al. [19] team building interventions, such as the ones reviewed here, are often judged subjectively by collecting data on participants perceptions of the interventions value. However, in order to fully understand if interventions are successful, more objective measures are needed. This is particularly true for interventions targeting psychological safety. Only three interventions reviewed here evaluated psychological safety as an outcome. While other interventions may have been effective in improving psychological safety, no measure was taken to verify this. There is a need to develop more objective ways of assessing the effectiveness of interventions targeting psychological safety. For example, the observational scheme developed by Hoenderdos et al. [50] provides a more objective measure of psychological safety. However, this measure has not yet been adapted for a healthcare context and further validation is needed. Future research should focus on developing more objective measures for assessing changes in psychological safety and its related behaviours in order to fully understand the effects of interventions.

#### Levels of intervention

Many of the interventions reviewed here were team level interventions, however, O’Leary [48] demonstrated the impact of factors at the organisational level by showing that it is difficult to develop psychological safety within organisations where shared decision making is not an organisational norm. In order for future research to develop effective interventions, they should target the organisational level, as well as the team level.

#### Who should participate in the intervention?

Studies in this review highlighted the importance and relevance of psychological safety, speaking up and voice behaviour within interdisciplinary teams [35, 36, 44, 47, 48]. This highlights the need for future interventions to address psychological safety, speaking up and voice behaviour across and between all disciplines. Studies also illustrated the need to involve team members in the development of interventions. Effective team building interventions ensure that team members contribute their knowledge of the team’s needs to inform the design of the intervention [19]. Four interventions in this review engaged with participants as part of the development stage of their intervention to ensure the intervention was grounded in reality of the participants work environment [43, 46–48]. However, other studies delivered pre-designed interventions that were not based on the needs of the participants. Adopting a co-design approach, where researchers and end-users collaborate in designing the intervention, can ensure that future interventions are tailored to teams needs. A study protocol published by Ward et al. outlines plans to work with key stakeholders, staff and patient representatives to co-design an intervention to create a culture of medical professionalism in relation to patient safety. Future research should ensure that participants are involved in the development stages on interventions, to ensure that the intervention is grounded in the team’s needs.

Lastly, team leaders play a key role in creating psychologically safe teams and should be involved in interventions [1]. In line with this, key stakeholders and leaders were involved in many of the studies reviewed here, either as a participant in the study or in facilitating the interventions. Leaders were involved in interventions through their behaviour facilitating psychological safety [37, 48] or through showing their support and commitment to the intervention [38, 40, 41, 47]. Since the search for this systematic review was conducted, a case study intervention focused on understanding voice and improving the response to disruptive behaviours has been published by Dixon-Woods et al. Leaders played a key role within this case study by becoming more open and willing to listen and to take staff concerns on board and by completing training in skills for encouraging voice and having difficult conversations. The interventions reviewed here, along with the recent paper by Dixon-Woods et al., highlight the important role leaders play in the success of interventions. This suggests that future research should ensure that key stakeholders and leaders are engaged with interventions in order to create a supportive environment that facilitates change.

### Strengths and limitations

In order to minimise the risk of publication bias, searches were conducted on academic and grey literature databases as well as through contacting experts. In addition, the eligibility of the included papers were independently screened by two reviewers.

Given the lack of interventions focused on psychological safety, the scope of this review was widened to included speaking up and voice behaviour. This allowed the review to gain a broader view of how interventions could be used to improve behaviours related to psychological safety. While these behaviours are strongly associated with psychological safety, the phenomenon of psychological safety is also associated with a variety of other concepts, including communication, decision making, team performance, team learning and divergent thinking. However, examining interventions which targeted each of these related concepts was beyond the scope of this review.

## Conclusion

This review is the first systematic review to examine interventions to improve psychological safety, speaking up and voice behaviour in healthcare teams. The mixed results found suggest a need to improve the effectiveness or efficacy, and measurement of these interventions. Longitudinal and multifaceted interventions may allow future studies to further investigate the efficacy or effectiveness of these interventions. In addition, the development and use of more objective measures may allow future studies to understand whether interventions are successful in improving psychological safety. Based on the identification of the successful elements of the interventions reviewed here, it is suggested that future intervention studies test the impact of these elements by involving end users in the design phase, target both group and organisational levels, ensure visible leader support and work across and within interdisciplinary teams.

## Supplementary information

**Additional file 1.** Search Strategy. Search strategies presented for each database searched.

**Additional file 2.** Data Extraction Template. The categories by which data was extracted from excluded studies are presented in the final template.

**Additional file 3.** Quality Assessment. The results of the quality assessment conducted are presented according to study design. The Critical Appraisal Skills Programme Qualitative Checklist, Cohort Study Checklist, or the Mixed Methods Appraisal Tool are presented.

**Additional file 4.** Summaries of included studies. Each table includes summary details of all interventions included in the review. Details are listed under the following titles: Author, Aims, Participants, Intervention Duration, Intervention Content, Methods of Evaluation, Key Findings.

## Data Availability

All data generated or analysed during this study are included in this published article [and its supplementary information files].

## Abbreviations

AHC: Abuse in Health Care
ARS: Audience Response System
CENTRE: Confidentiality, Equal airtime, Non-Judgemental (respectful) listening, Timeliness, Right to pass, Engaged
CNO: Chief Nursing Officer
CPPS: Centre for Professionalism and Peer Support
CRM: Crew Resource Model
CUS: Concerned, Uncomfortable, Stop
DESC: Describe, Express, Suggest, Consequences
ED: Emergency Department
NRP: Neonatal Resuscitation Program
NRP: Neonatal Resuscitation Program
OB/GYN: Obstetrics/gynocology
OCLC: Online Computer Library Center
PARIHS: Promoting Action of Research Implementation in Health Service
PRISMA: Preferred Reporting of Items for Systematic Reviews and Meta-Analysis
SBAR: Situation, Background, Assessment, Recommendation
T1/2/3: Time 1/2/3
Team STEPPS: Team Strategies and Tools to Enhance Performance and Patient Safety
WHO: World Health Organisation
