# Supplementary material for: A systematic review exploring the content and outcomes of interventions to improve psychological safety, speaking up and voice behaviour
Source: BMC Health Serv Res. 2020 Feb 10;20:101. doi: 10.1186/s12913-020-4931-2 (PMC7011517; doi:10.1186/s12913-020-4931-2)
Supplement: Supplementary file 1 — Additional file 1. Search Strategy. Search strategies presented for each database searched. [file 12913_2020_4931_MOESM1_ESM.docx]

**Detailed Search Strategies**

***PsychInfo and ABI Inform search string***

AB,TI(“Psychological* safe*” OR “speak* up” OR voic* OR silen*)

***PubMed search string***

((("Psychological* safe*"[Title/Abstract] OR "speak* up"[Title/Abstract] OR voic*[Title/Abstract] OR silen*[Title/Abstract])) AND ( "1999/01/01"[PDat] : "2018/12/31"[PDat] ))

***Academic search complete search string***

[AB “Psychological* safe*” OR “speak* up” OR voic* OR silen*](https://web.b.ebscohost.com/ehost/breadbox/search?term=AB%20%E2%80%9CPsychological%2A%20safe%2A%E2%80%9D%20OR%20%E2%80%9Cspeak%2A%20up%E2%80%9D%20OR%20voic%2A%20OR%20silen%2A&sid=00f52e19-e7ab-4f85-84fe-502c2182a80a%40pdc-v-sessmgr06&vid=3)
