# Supplementary material for: A systematic review exploring the content and outcomes of interventions to improve psychological safety, speaking up and voice behaviour
Source: BMC Health Serv Res. 2020 Feb 10;20:101. doi: 10.1186/s12913-020-4931-2 (PMC7011517; doi:10.1186/s12913-020-4931-2)
Supplement: Supplementary file 2 — Additional file 2. Data Extraction Template. The categories by which data was extracted from excluded studies are presented in the final template. [file 12913_2020_4931_MOESM2_ESM.docx]

**Data Extraction Template**

| Review title or ID |  |
| --- | --- |
| Author(s) |  |
| Date published |  |
|  | |

# General Information

| Date form completed *(dd/mm/yyyy)* |  |
| --- | --- |
| Reference citation |  |
| Study author contact details |  |
| Publication type |  |

# ***Characteristics of included studies***

## Methods

|  | Descriptions as stated in report/paper | | Location in text or source |
| --- | --- | --- | --- |
| Aim of study |  | |  |
| Design |  | |  |
| Duration of study |  | |  |
| Details of intervention (Mode of delivery, duration and content) |  | |  |
| Theoretical basis |  | |  |
| Variables/measures of interest |  | |  |
| Ethical approval needed/ obtained for study | Yes No Unclear |  |  |

## Participants

|  | Description | | Location in text or source |
| --- | --- | --- | --- |
| Population description |  | |  |
| Setting  *(including location and social context)* |  | |  |
| Inclusion criteria |  | |  |
| Exclusion criteria |  | |  |
| Method of recruitment of participants |  | |  |
| Informed consent obtained | Yes No Unclear |  |  |
| Baseline imbalances |  | |  |
| Withdrawals and exclusions |  | |  |
| Age |  | |  |
| Sex |  | |  |
| Race/Ethnicity |  | |  |
| Other relevant sociodemographics |  | |  |
| Subgroups measured |  | |  |
| Subgroups reported |  | |  |
| Notes: | | | |

## ***Outcomes***

Outcome 1

|  | Description as stated in report/paper | | Location in text or source |
| --- | --- | --- | --- |
| Outcome name |  | |  |
| Outcome definition |  | |  |
| Unit of measurement  (if relevant) |  | |  |
| Scales: upper and lower limits *(indicate whether high or low score is good)* |  | |  |
| Is outcome/tool validated? | Yes No Unclear |  |  |

# ***Other information***

|  | Description as stated in report/paper | Location in text or source |
| --- | --- | --- |
| Key conclusions of study authors |  |  |
| References to other relevant studies |  |  |
| Correspondence required for further study information (from whom, what and when) |  | |
