# Supplementary material for: A systematic review exploring the content and outcomes of interventions to improve psychological safety, speaking up and voice behaviour
Source: BMC Health Serv Res. 2020 Feb 10;20:101. doi: 10.1186/s12913-020-4931-2 (PMC7011517; doi:10.1186/s12913-020-4931-2)
Supplement: Supplementary file 3 — Additional file 3. Quality Assessment. The results of the quality assessment conducted are presented according to study design. The Critical Appraisal Skills Programme Qualitative Checklist, Cohort Study Checklist, or the Mixed Methods Appraisal Tool are presented. [file 12913_2020_4931_MOESM3_ESM.docx]

**QUALITY ASSESSMENT CHECKLISTS**

Results of CASP Qualitative Checklist

| Author, Year | Statement of aims | Qualitative methodology appropriate | Research design appropriate | Recruitment strategy appropriate | Data collection appropriate | Relationship between researcher and participant considered | Ethical issues considered | Data analysis rigorous | Statement of findings | Is research valuable | Outcome of checklist (Yes/Can’t tell/ No) |
| --- | --- | --- | --- | --- | --- | --- | --- | --- | --- | --- | --- |
| Swahnberg & Wijma, 2012 | Yes | Yes | Yes | Yes | Can’t tell | Can’t tell | Yes | Yes | Yes | Yes | 8/2/0 |
| Brown and McCormack, 2016 | Yes | Yes | Yes | Yes | Yes | Yes | Yes | Yes | Yes | Yes | 10/0/0 |
| O’Leary., 2016 | Yes | Yes | Yes | Yes | Yes | Yes | Yes | Yes | Yes | Yes | 10/0/0 |

Results of Mixed Methods Appraisal Tool (MMAT) Quantitative descriptive

| Author, Year | Clear objective | Data relevant to addressing objectives | Qualitative data sources relevant to objectives | Qualitative data analysis relevant to objectives | Consideration given to how findings relate to context | Consideration given to how findings relate to researchers’ influence | Sampling strategy relevant to address quantitative research question | Sample representative of population | Quantitative measure appropriate | Acceptable response rate* | Mixed methods design relevant to objective | Integration of qualitative and quantitative relevant to objective | Limitations of integrating findings considered | Outcomes of checklist (Yes/Can’t tell/No) |
| --- | --- | --- | --- | --- | --- | --- | --- | --- | --- | --- | --- | --- | --- | --- |
| Pian-Smith et al., 2009 | Yes | Yes | Yes | Yes | Yes | Can’t tell | Yes | Yes | Yes | Yes | Yes | Yes | Can’t tell | 11/2/0 |
| Shapiro et al., 2014 | Yes | Yes | Yes | Can’t tell | Yes | Can’t tell | Can’t tell | Yes | Yes | Can’t tell | Yes | Yes | Can’t tell | 8/5/0 |
| Dufresne., 2007 | Yes | Yes | Yes | Yes | Yes | Yes | Yes | Yes | Yes | Can’t tell | Yes | Yes | Yes | 12/1/0 |
|  |  |  |  |  |  |  |  |  |  |  |  |  |  |  |

Results of Mixed Methods Appraisal Tool (MMAT) Quantitative non-randomised

| Author, Year | Clear objective | Data relevant to addressing objectives | Qualitative data sources relevant to objectives | Qualitative data analysis relevant to objectives | Consideration given to how findings relate to context | Consideration given to how findings relate to researchers’ influence | Participants recruited to minimise bias | Measurements appropriate for exposure/intervention and outcomes | Comparable groups | Complete outcome data (80%+)/response rate (60%+)/ follow up rate | Mixed methods design relevant to objective | Integration of qualitative and quantitative relevant to objective | Limitations of integrating findings considered | Outcomes of checklist (Yes/Can’t tell/No) |
| --- | --- | --- | --- | --- | --- | --- | --- | --- | --- | --- | --- | --- | --- | --- |
| Sayre et al., 2012 | Yes | Yes | Yes | Yes | Yes | Can’t tell | Yes | Yes | Yes | Can’t tell | Yes | Yes | Can’t tell | 10/3/0 |
| O’Connor et al., 2013 | Yes | Yes | Yes | Can’t tell | Can’t tell | Can’t tell | Yes | Can’t tell | Yes | Yes | Yes | Can’t tell | Can’t tell | 7/6/0 |
|  |  |  |  |  |  |  |  |  |  |  |  |  |  |  |

Results of Mixed Methods Appraisal Tool (MMAT) Quantitative randomised control trial

| Author, Year | Clear objective | Data relevant to addressing objectives | Qualitative data sources relevant to objectives | Qualitative data analysis relevant to objectives | Consideration given to how findings relate to context | Consideration given to how findings relate to researchers’ influence | Clear description of randomisation | Clear description of blinding | Complete outcome data (80%+) | Low withdrawal (below 20%) | Mixed methods design relevant to objective | Integration of qualitative and quantitative relevant to objective | Limitations of integrating findings considered | Outcomes of checklist (Yes/Can’t tell/No) |
| --- | --- | --- | --- | --- | --- | --- | --- | --- | --- | --- | --- | --- | --- | --- |
| Raemer et al., 2016 | Yes | Yes | Yes | Yes | Yes | Can’t tell | Yes | Yes | Yes | Yes | Yes | Yes | Can’t tell | 11/2/0 |
| Thomas et al., 2007 | Yes | Yes | Yes | Yes | Yes | Can’t tell | Yes | Yes | Yes | Yes | Yes | Yes | Can’t tell | 11/2/0 |
|  |  |  |  |  |  |  |  |  |  |  |  |  |  |  |

Results of CASP Cohort Study Checklist

| Author, Year | Focused issue addressed | Acceptable recruitment | Exposure measured to minimise bias | Outcome measured to minimise bias | Confounding factors identified | Design/ analysis considers all important confounding variables | Follow up of subjects complete enough | Follow up of subjects long enough | Results believed | Can results be applied to local population | Results fit with other evidence | Outcome of checklist (Yes/ Can’t tell /No) |
| --- | --- | --- | --- | --- | --- | --- | --- | --- | --- | --- | --- | --- |
| Johnson & Kimsey 2012 | Yes | Yes | Yes | Can’t tell | Can’t tell | Can’t tell | Yes | Can’t tell | Yes | Can’t tell | Yes | 6/5/1 |
| Cave et al., 2016 | Yes | Can’t tell | Can’t tell | No | Can’t tell | Can’t tell | Can’t tell | Can’t tell | Can’t tell | Can’t tell | Can’t tell | 1/8/2 |
| Coyle et al., 2005 | Yes | Can’t tell | Yes | Yes | Can’t tell | Can’t tell | Yes | No | Yes | Yes | Yes | 7/3/1 |

Results of CASP Case control Study Checklist

| Author, Year | Focused issue addressed | Appropriate method used | Acceptable recruitment of cases | Acceptable recruitment of controls | Exposure accurately measured | Groups treated the same | Taken account of confounding factors | Results believed | Can results be applied to local population | Results fit with other evidence | Outcome of checklist (Yes/ Can’t tell /No) |
| --- | --- | --- | --- | --- | --- | --- | --- | --- | --- | --- | --- |
| Ginsburg & Bain., 2017 | Yes | Yes | Yes | Yes | Yes | Yes | Can’t tell | Yes | Yes | Yes | 9/1/0 |
|  |  |  |  |  |  |  |  |  |  |  |  |
|  |  |  |  |  |  |  |  |  |  |  |  |
