# Supplementary material for: A systematic review exploring the content and outcomes of interventions to improve psychological safety, speaking up and voice behaviour
Source: BMC Health Serv Res. 2020 Feb 10;20:101. doi: 10.1186/s12913-020-4931-2 (PMC7011517; doi:10.1186/s12913-020-4931-2)
Supplement: Supplementary file 4 — Additional file 4. Summaries of included studies. Each table includes summary details of all interventions included in the review. Details are listed under the following titles: Author, Aims, Participants, Intervention Duration, Intervention Content, Methods of Evaluation, Key Findings. [file 12913_2020_4931_MOESM4_ESM.docx]

**TABLES**

Table 1.

Summary of Education and Simulation interventions

| Author | Aims | Participants | Underpinning Theory | Intervention Duration | | Intervention Content | Methods of Evaluation | Key Findings |
| --- | --- | --- | --- | --- | --- | --- | --- | --- |
| Pian-Smith et al., 2009 | To determine whether an intervention focused on joint responsibility to speak up and the two-challenge rule can improve frequency and effectiveness of “speak up” behaviour. | 40 trainee anaesthesiologists. | Crisis Resource Model (CRM), Model I and Model II conversation patterns and Advocacy and Inquiry challenges. | 30-35-minutes. | Participants presented with one of two simulated scenarios with an opportunity to challenge a resident’s attending faculty anaesthetist, an attending faculty surgeon and circulating nurse. Followed by a debriefing session including discussion and self-reflection. Shared responsibility for speaking up and the two-challenge rule^[[1]](#footnote-1)^ were introduced and the “advocacy-inquiry” communication approach was demonstrated. Participants were given the opportunity to apply learnings in the second simulated scenario. | | Scenarios were video recorded and the language used were coded independently by two investigator anaesthesiologists (blind to which scenario they were coding). Codes used: said nothing, made an oblique statement, made an advocacy statement or an advocacy-inquiry statement. Scores after debriefing and instruction were compared with those before. | Participants used an increased frequency of advocacy and inquiry after the debriefing (27% pre-training to 65% post-training).  Quality of language directed towards faculty anaesthesiologists and faculty staff improved (p=0.0004). Advocacy-inquiry language use increased from pre-training (16%) to post-training (72%).  Improved challenges towards attending faculty surgeons (p=0.002). Advocacy-inquiry language use increased from pre-training (29%) to post-training (67%).  The quality of challenges verbalised to nurses were not improved over all (P=0.84). |
| Raemer et al 2016 | Investigate if a simulation-based intervention would improve speaking up behaviours and identify hurdles and enablers to speaking up. | Non-trainee anaesthesiologists.  Randomly assigned to intervention (n=35) or control (n=36) groups. | Team training for speaking up- TeamSTEPPS. | 50 minutes. | Workshop presented patient safety rationale for speaking up, conversational skills, the “two challenge rule” and a role play exercise. Participation in experimental scenario with opportunities to speak up to a surgeon, a circulating nurse and an anaesthesiologist colleague. Followed by a debriefing session. | | Workshop conducted with experimental group before the simulated scenario. The control group took part in the scenario before the workshop. Simulated scenarios were video recorded and coded. The debriefing sessions were coded for hurdles and enablers to speaking up. | No statistically significant difference in speaking up behaviours between groups. Limited frequency and quality of speaking up by all participants. |
| Thomas et al 2007 | Incorporate teamwork skills and information about human error into a 1-day Neonatal Resuscitation Program (NRP) and measure the effects of teamwork during simulated resuscitations. | 49 interns randomised to intervention and control group. | Teamwork and communication in neonatal resuscitation. | 1 day training course. | Educational component addressed human error, inquiry, information sharing, assertion, evaluation of plans, workload management and vigilance. Role plays and video clips illustrated concepts and skills.  Interns received the full NRP course. After a skill was taught, interns practiced it on low-fidelity mannequins. The intervention group were prompted to practice the team behaviours taught.  Each intern led a team in a mock resuscitation on low-fidelity mannequins. | | Resuscitations were video recorded and behaviours were coded by two blinded observers. Inquiry information sharing, assertion and evaluation of plans were identified in verbalizations of team members. Vigilance and workload management were measured as percent time in that particular state. Cohen’s k for each teamwork behaviour was calculated and the definition of each behaviour was clarified. | Interns in the intervention group exhibited more frequent information sharing, inquiry and assertion than the control group.  Evaluation of plans was observed once in the intervention group and not at all in the control group.  Vigilance and workload management were practiced throughout the entire simulation intervention teams (100% vigilance, 88% workload management). Only 20% of the control teams were vigilant and managed their workload throughout the entire simulation. |
| Ginsburg & Bain 2017 | Evaluate a multifaceted intervention designed to promote speaking up and improve teamwork climate. | Emercency department: 102 staff (baseline/T1), 82 staff (3 months post baseline/T2), 105 staff (7 months post baseline).  Intensive care unit (control unit): 31 (T1), 38 (T2), 30 (T3). | Implementation science: multi-faceted intervention, engaging stakeholders and interactive elements.  Leadership: leader facilitated initiatives to improve trust or establish new cultural norms  Experiential education: Role playing, reflection, interactive over didactic learning. | Not stated | 1) Workshop introduced organisation Accountability Framework and scripted conversation and listening enablers. Role playing simulation gave participants opportunity to use these tools. Followed by debriefing session and exit survey.  2) Six weekly briefings conducted after the workshop. Discussion was facilitated by interprofessional practice lead and focused on how the ED might improve teamwork climate scores.  3) 10 minute staff huddles were conducted four times daily. Staff encouraged to provide input on any aspect of ED operations. One to one meetings to review performance and obtain feedback from staff about the department. The staffs suggestions were acted upon. | | Teamwork climate version of the Teamwork Climate Survey. The scale includes 6 items of a 5-point Likert-scale. Items addressed broad teamwork issues as well as speaking up. Participates completed an Exit Survey with ten questions that reflect educational outcomes at level 1 of Kirkparticks framework. | Workshop evaluation: Workshop relevant to staff’s day to day work. More than 80% of staff were satisfied with overall experience and would recommend participation to others. 93% found opening scenarios valuable while only 40% agreed the small group role playing simulation exercise was valuable.  Change in teamwork climate scores.  Overall improvement in teamwork climate perceptions for intervention unit but not control unit. |
| Dufrense., 2007 | Capture the effects of naturalistic variability in team debriefing leader behaviour on team psychological safety and learning behaviours. | Forty ad hoc anaesthesia teams, 227 resident anaesthesiologists. | Team and organisational learning, crisis management, debriefing, action science and analogical learning. Focus on effects of leaders within these literatures. | 21-90 minutes. | After completing a simulated medical critical incident, participants were guided through a group reflection on the experience by a faculty anaesthesiologist who did not participant in the critical incident. | | Video taped and content analysed team debriefings. Questionnaires collected control variables (age, extroversion) and psychological safety scares. | Significant negative effects of leaders’ use of transparent evaluations and balancing of inquiry and advocacy statements on team psychological safety. Worse balancing and less transparent evaluative statements led to greater team psychological safety.  When team debriefing leaders made negative evaluative statements, there as a resulting lower level of psychological safety.  Significant positive effect of the team debriefing leaders’ use of analogy on team psychological safety and team learning behaviours. |

Table 2.

Summary of Education and Leadership Video Interventions

| Author | Aims | Participants | Underpinning Theory | Intervention Duration | | Intervention Content | Methods of Evaluation | Key Findings |
| --- | --- | --- | --- | --- | --- | --- | --- | --- |
| O’Connor et al., 2013 | Conduct training program to a) Demonstrate the importance of speaking up for patient safety and quality of care b) Show that attending physicians support speaking up, c) Provide speaking up. | 110 interns. | CRM training  Speaking up. | 90 minutes. | Brief presentation on human factors and human error in healthcare. Review of nontechnical skills required of effective interns. Instruction on communication techniques adapted from aviation.  Presentation of four attending physicians’ film clips describing challenging situations they faced as interns in which their communication and assertiveness skills were challenged, their reflections on what they could have done better, followed by a group discussion on speaking up. | | Survey measuring participants reactions, learning and behaviour. 50 participants from training group and 35 from control group completed standardised patient exercises^[[2]](#footnote-2)^. | Positive reaction to the training programme.  Knowledge: Significant improvement post training group. Experimental group had higher number of correct answers compared to control group.  Attitudes to speaking up: No significant effect on speaking up about stress, significantly more positive attitudes towards speaking up to seniors post training. No significant difference between training groups and control group.  Training had no significant effect on behaviours during standardised patient exercises. |
| Sayre et al 2012 | Evaluate an intervention designed to develop speaking up behaviours among nurses and to improve their behaviour in situations where patient safety is in jeopardy. | 58 registered nurses in intervention group with 53 taking part in the posttest.  87 registered nurses in control group with 51 taking part in posttest. | Speaking up- defined as voice to make specific information that is privately held know to someone with positional power or authority to take action. | 5-6 hours. | Video shown of the chief nursing officer (CNO) and a physician leader from the hospital expressing their expectation that and support for nurses speaking up. Participants discussed organisational obstacles for speaking up. These were shared with the facility CNO and nurse leaders after the study.  In groups, nurses discussed obstacles that prevented speaking up. They generated an action plan of what they would do differently to improve speaking up. They exchanged contact details and agreed to meet and support one another. | | Surveys measuring demographics and speaking up.  Individual List of Nurse Behaviours. Participants watched a video 5 real-life vignettes showing how a failure of the registered nurse to speak up led to negative patient outcomes. Participants asked to list any behaviours which they would have used in the situation. A panel of experts rated the behaviour responses at time 1 and time 2 on a scale of 1 to 5 for strength of speaking up. | The intervention group showed a significant difference in mean speaking up scores from baseline to post-test (p=.0001). There was no significant change in the control group (p=.68)  The intervention group showed statistically significant difference in mean scores on the list of individual nurse behaviours from baseline to post-test (p=.0001). No difference was found for the control group (p=.27). |

Table 3.

Summary of Education and Case Study Interventions

| Author | Aims | Participants | Underpinning Theory | Intervention Duration | | Intervention Content | Methods of Evaluation | Key Findings |
| --- | --- | --- | --- | --- | --- | --- | --- | --- |
| Johnson & Kimsey,2012 | Address staff members’ discomfort with speaking up and provide tools to enhance communication during difficult or unusual events. Invite open communication in an environment where challenging authority is welcomed. | 809 healthcare clinicians who were members of anesthesia, surgery and obstetrics/gynocology (OB/GYN). Ranging from physician level to allied healthcare personnel. | Communication enhancement  Crew Resource Manegement (CRM)  TeamSTEPPS. | 3 hours. | Videos shown portraying risk and error followed by discussion with audience.  Discussions focused on need for shared purpose and common goals, recognising the interdependence, being accountable, and leadership.  Team communication: Discussion on experiences, barriers and enablers of good communication. Techniques introduced: situational awareness, SBAR^[[3]](#footnote-3)^ and callout and check-back technique introduced, WHO Surgical safety checklist. Discussion on conflict and conflict resolution, introduced DESC^[[4]](#footnote-4)^ approach to conflict resolution and assertion techniques (CUS, 2 challenge rule and the “Stop the Line)^[[5]](#footnote-5)^. | | Audience response system (ARS) remote given to attendees. Baseline information collected for demographics, attendees’ comfort with speaking out for patient safety. Post-training participants were given an evaluation survey and measures of their understanding of the course content were collected. | Post course evaluations: 78% believed that they were better able to question decisions or actions of those with more authority. 75% said they were no longer afraid to ask questions when something did not seem right.  Feedback during course: 25% thought the communication tools were most useful, 21% thought the videos were most useful and 14% thought that review of the importance of safety and having OR process in place were most useful.  Marked decrease in the number of near misses and sentinel events requiring Root Cause Analysis post intervention. |
| Coyle et al., 2005 | Evaluate a patient safety educational program for its effectiveness in improving attitude and behaviour related to medical event reporting. | 30 graduate trainees in an ambulatory care setting. | Medical event reporting  Patient safety education. | Seven 1 hour conference. | Each conference had an educational theme and discussion of an ambulatory case event from within the study clinic. Group discussions were facilitated by faculty members. Each group completed a structure worksheet on the event followed by a large group discussion. Lastly, there was a case summary and written materials were distributed. | | Attitudes and behaviour related to medical event reporting assessed by a self-administered 5 item questionnaire at baseline and at 6 month follow up. Barriers to medical event reporting assessed by self-administrated 5 item questionnaire at 6 months follow up. | No difference found in attitude and behaviour towards medical event reporting. However, the number of conferences attended by participants were significantly correlated with change in medical event reporting attitude and behaviour score at 6 months follow up. With those who participated more showing positive changes in attitude and behaviours.  Most frequently cited barrier was lack of time to report due to other clinic duties. Other major barriers were extra paper work and concern about career and personal reputation and not recognising any medical events to report. |
| Shapiro et al., 2014 | To educate a hospital community regarding professionalism and a process for managing professionalism lapses.  To encourage a culture that values and promotes mutual respect, trust and teamwork. | 793 bed tertiary care facility serving as a major teaching hospital of Harvard Medical School. Physician and scientist faculty and individuals from 13 major specialty departments 1,287 physicians and scientists completed the session from October 10, 2010 to December 31, 2012. | Unprofessional behaviours: Diminish and destroy relational trust (between healthcare team members and between healthcare professionals and their patients).  Professionalism: Any intent, action or words that foster trustworthy relationships. | Educational program: 1.5 hours  Code of conduct: ongoing. | Leadership support and the code of conduct: Centre for Professionalism and Peer Support (CPPS). Engaged leaders within the hospital. Developed a Code of Conduct to provide an opportunity for staff to restate their commitment to one another and to patients.  Mandatory educational programs included videotaped vignettes and workbooks. Open discussion on professional behaviour and the responsibility of colleagues and bystanders to speak up. A series of voluntary interactive workshops to enhance communication and conflict management skills offered.  Process for managing professionalism concerns: Confidential intake of concerns, assessment process (coaching, interviews or investigation of reported behaviour) and remediation and monitoring. | | Interviews conducted.  Evaluation survey: Indicate professional role and provide an assessment of: Whether the objectives of the session were met, whether the session improved their understanding of professionalism and whether the session enhanced their professional practice. Written comments on: important lessons, strength of the session and improvements that could be made. | Training sessions were rated positively for objectives being achieved, awareness being increased and sense that the session will enhance professional practice in both 2011 (means; 1.5, 1.7, 1.8) and 2012 (means; 1.6, 1.7. 1.8) sessions. Comments indicated acceptance of a personal role in ensuring a culture of professionalism and appreciation that a functional system exists to manage unprofessional behaviours.  Number of reports regarding professional behaviour increased each year, 25 in 2010, 51 in 2011 and 71 in 2012. In total, reports were received from 201 physicians/scientists and 8 healthcare teams.  Interventions used following reports: Feedback conversation with the person who had displayed unprofessional behaviour and their supervisory physician. Some completed behavioural coaching. Interviews were completed with relevant individuals to assess behavioural outcomes which revealed substantive change in behaviour of multiple individuals. |

Table 4.

Summary of Educational Workshop Intervention

| Author | Aims | Participants | Underpinning Theory | Intervention Duration | | Intervention Content | Methods of Evaluation | Key Findings |
| --- | --- | --- | --- | --- | --- | --- | --- | --- |
| Cave, Pearson, Whitehead & Rahim-Jamal (2016) | Describes an innovative tool developed to facilitate psychological safety in a variety of group environments. | 17 group leaders from healthcare groups. | Challenges of fostering psychological safety in healthcare teams. | Not stated. | Agreements to promote psychological safety. Psycho-education regarding use the agreements provided and definitions are reviewed. The group is asked if agreements need to be modified to suit their needs, changes are clearly defined. Newcomers must have an opportunity to agree to guidelines and they must be revised regularly. | | Question regarding the use of CENTRE asked on a 10 point Likert scale ranging from “not helpful at all” to “extremely helpful”. | Aggregate ranking for 9.1 suggesting that the group did find it helpful to create guidelines of how to work together. |

Table 5.

Summary of Non-Educational Interventions

| Author | Aims | Participants | Underpinning Theory | Intervention Duration | | Intervention Content | Methods of Evaluation | Key Findings |
| --- | --- | --- | --- | --- | --- | --- | --- | --- |
| Swahnberg & Wijma 2012 | Understand staff’s perception of abuse in health care (AHC) after an intervention based on ‘Forum Play’. | 74 healthcare staff. 21 interviewed at baseline and 10 interviewed after completing the workshop. | Abuse in Healthcare (AHC): Failed health care encounters in which patients feel abused and suffer.  Role play: A technique to address interpersonal components of quality of care during interventions. | 17 half day workshops- each participant took part in at least 1 | Forum Play aims to create a safe place fot acting out new solutions to difficult situations. It is hoped that participants gradually feel brave enough to transfer their experiences into real life. Staff role-played AHC events which typically included a bystander who was not intervening but felt that they should. The scene was repeated until it was clear to everyone what the moral conflict was and who was suffering the moral conflict. When the scene was played again, the audience was encouraged to intervene by entering the scene to change the outcome by taking on the role of the person who suffered the moral conflict. | | Interviews to understand staffs perceptions of AHC. | Before the intervention staff reported detached perceptions of AHC. AHC had a taboo status at the clinic. It was recognised as a transgression of ethical principles but staff often justified it. They had limited their ability to act against AHC.  Post-intervention, acting against AHP had become an imperative. The taboo status of AHC had been broken. It was present more often in daily conversations and it had become a shared problem. Participants emphasised the role played by bystanders in noticing and stopping or preventing AHC. Most informants reported that speaking up about AHC was an option for them and they showed strong emotional engagement through their examples of AHC, showing an awareness of their responsibility in relation to AHC. |
| Brown & McCormack 2016 | Outline how psychologically safe spaces can be created through holistic facilitation, to enable more effective person-centred cultures. | Two wards of an abdominal surgical unit.  The lead nurse, medical team (n=3), ward managers (n=2), nursing staff (n=48). | Promoting action on Research Implementation in health service (PARIHS) conceptual framework (Kitson et al., 1998):  Integrated PARIHS framework (Harvey & Kitson, 2015). | Not stated. | Holistic facilitation: Working with and supporting practitioners to enable them to explore basic assumptions and improvements in practice.  The facilitator/lead researcher worked with participants, as co-researchers, to develop their practice. The facilitator role modelled supportive behaviours, was accessible, listened attentively, asked facilitative questions, was tenacious and encouraged participants to take action. Using critical reflection, the facilitator encouraged them to pinpoint the problems they experienced daily and explore the assumptions they made about their practice. With facilitator support, individuals and team planned and implemented agreed actions. Actions taken were evaluated through on-going data collection and analysis. | | Facilitated reflective sessions involved periods of time for up to 5 members of the nursing team to critically reflect on issues they faced in their practice. 26 formal session were conducted and 26 ad hoc sessions. The lead nurse and ward managers took part in one to one facilitation with the leader researcher/facilitator (27 sessions total). Data were gathered using flip charts and were shared with the team to ensure collective understanding. Lead researcher reflective journal: systematically recorded empirical events and difficulties or successes at the end of all facilitation sessions. | Support: The facilitator role modelled ways the team could support one another and was available to offer guidance when needed. This gave ward leaders the psychological safety to address the practice issues and deliver person centred care in more positive ways.  Oppressed behaviours: The holistic facilitation created psychologically safe spaces that supported nurses to explore and learn about their oppressed behaviours. This helped nurses to consider was in which they could communicate more effectively and openly with the multidisciplinary team about differences in opinion and impact on patient care.  Leadership: Through facilitated reflection, the ward leaders become more supportive in seeking ways to address leadership issues and call staff to account. They became more aware of the important role they played in setting the culture in their unit. This allowed carefully negotiated and trusting partnerships to build and for experiences and knowledge to be shared as a resource to help solve problems and take appropriate action. The psychological safety created helped the ward leaders to challenge negative behaviours. |
| O’Leary., 2016 | To explore the importance of team psychological safety in the development of two interprofessional teams. | Project teams at two residential care facilities for older people located in Ireland.  Oakwood Heights: 13 care providers joined the team.  Sheltered Cove: 11 care providers and 1 client joined the team. | Team psychological safety: An atmosphere within a team where individuals feel comfortable engaging in discussion and reflection without fear of censure. | 9 months. | The teams met approximately one a month. Each team engaged in cycles of action research where potential improvements in practice were discussed, implemented and evaluated. Changes included the development of a pain assessment tolls, setting up a family support group, setting up staff training sessions and the development of information booklets.  Leadership model used within meetings was based on listening actively, using positive language, treating team members with respect, giving positive and constructive feedback, seeking input from all team members and encouraging group reflection in meetings. | | Field notes: recorded data generated in both formal and informal settings and to ensure that actions, reflections and learning are recorded. Semi-structured interviews, group discussions and questionnaires were also used to collect qualitative data. Data were analysed using cycles of analysis activity involving data reductions, display, conclusions drawing and verification. | Team psychological safety was developed differently the teams.  *Power sharing*: Oakwood Heights: Individuals started asserting themselves in meetings, engaging in shared decision-making, volunteering and assigning responsibility. Sheltered Cove: Team psychological safety did not develop to a point where team members felt comfortable sharing power.  *Knowledge co-generation:*Oakwood Heights: participants felt secure enough to verbalise their tacit knowledge and this led to co-generation of knowledge about team roles.  Sheltered Cove: confusion about role boundaries were never fully resolved. Co-generation of knowledge did not occur.  *Organisational norms:* Oakwood Heights: shared decision making was evidenced by high input from care assistants. Sheltered Cove: care assistants self-censored and did not contribute. They stopped attending meetings after meeting 3 due to feelings of low power which made them wary of engaging in joint decision-making.  *Stability in team membership*: Oakwood Heights: Core group of six members attended most of the meetings and they developed interpersonal relations and trust. Sheltered Cove: Higher change over in team members. By meeting 4 that a core group of three members emerged -began to engage in more open and honest discussion. |

1. A tool that places responsibility to speak up on all team members by allowing one member to automatically assume the duties of another member if they fail to respond to two consecutive challenges. This technique is used when one has observed an action that makes them uncomfortable, they first speak up by challenging the action from a position of curiosity; if this gets no response, they speak up from a position of concern and finally, they report their concerns to someone in a position of authority. [↑](#footnote-ref-1)
2. These exercises were adapted from the programme for medical innovations and research in New York University Langone Medical centre and involved the interns reading an introduction text which sets the scene for the scenario. They then proceed to manage the situation presented in the scenario with an actor playing the person with whom they are interacting. The scenario was observed by either a physician or psychologist who rated the interns’ behaviour on a rating scale designed for each scenario. [↑](#footnote-ref-2)
3. The SBAR (Situation-Background-Assessment-Recommendation) technique offers a framework for engaging in critical conversations in order to communicate critical information on the patient’s situation and condition to different team members. [↑](#footnote-ref-3)
4. The DESC approach involved describing the specific situation, expressing concerns about the action, suggest other alternatives, and stating the consequences. This method can be used by team members in order to reach a consensus about how to resolve the conflict. [↑](#footnote-ref-4)
5. In the CUS approach, a team member states, “I am concerned.” If the situation escalates, they state, “I am uncomfortable.” If the safety threat continues, the team member says, “Stop, there is a safety issue.” This last assertion is also known as the “Stop the line” technique. [↑](#footnote-ref-5)
